# The Best Summer Beverage

**Published:** 1878-09

**Authors:** 


THE BEST SUMMER BEVERAGE, is cold water, ice cold, if you please, but by all means grasp the glass by the hand, take a swallow at a time, remove the glass from the lips for a few seconds then take another swallow; in this way it will be found that the thirst will be thoroughly satiated before half the water has been taken, whereas if it had been swallowed continously, the whole contents would not have satisfied the thirst.

As many persons have dropped dead from drinking greedily of water not ice cold, when very much heated and fatigued, this precaution may save many a life.

Butter-milk is another admirable summer beverage. Its acidity cools the system and acts slightly on the liver, and thus promotes the passing of the bile and other impurities from the body.

SUSAN PIERSON, of Bridgehampton, Long Island, died **Feb. 24th,** " in her 72d year

Her case was peculiar ; it is probable it has no parallel. For more than fifty years she did not set her foot upon the floor, and in all that time did not sit upright in bed. One year of that time was spent at a neighbor's house, with which exception the extent of her travels in fifty years was from one corner of her room to another, once a week, in some strong man's arms. This change was always attended with an almost entire loss of voice, from which she did not recover until after a night's repose.

